# Polymeric Materials in Energy Conversion and Storage

**DOI:** 10.3390/polym16223132

**Published:** 2024-11-10

**Authors:** Vineet Kumar, Md Najib Alam

**Affiliations:** School of Mechanical Engineering, Yeungnam University, 280, Daehak-ro, Gyeongsan 38541, Republic of Korea; vineetfri@gmail.com

## 1. Introduction

Energy conversion and storage devices based on polymeric materials are emerging as a promising avenue for renewable power sources. These features are attributed to their versatility, tunable properties, and ease of processing for polymer-based energy materials [1]. Due to their versatile nature, these polymeric materials are currently used in a wide range of applications, such as batteries, fuel cells, solar cells, nanogenerators, and supercapacitors [2]. Moreover, the ability to engineer these materials at the molecular level provides significant advantages in optimizing the performance, efficiency, and longevity of energy systems. Therefore, these materials emerge as promising renewable power sources with high efficiency and durability [3]. There are various advantages to using polymer materials in energy harvesting applications. For example, their unique properties—such as flexibility, lightweight nature, chemical stability, and ease of processing—make them attractive for energy applications. Additionally, these polymers can be chemically modified to enhance specific functionalities like conductivity, ion transport, or mechanical strength, depending on the need [4].

There are various energy conversion applications for polymer-based energy generation systems. Some important applications include solar cells, fuel cells, and energy generators [5,6]. Solar cells utilize polymer-based organic photovoltaic cells that harvest light energy and convert it into electrical energy. For example, organic semiconductors made from conjugated polymers can absorb sunlight and generate electrical energy. These polymer-based harvesting materials are promising due to their lightweight nature, flexibility, low cost, and ease of production via 3D and 4D printing [7]. In addition, fuel cells use polymer-based Nafion membranes (also known as proton exchange membranes) to conduct protons from the anode to the cathode. These membranes are crucial because they separate the fuel and oxidant while allowing ion transport, which is essential for the electrochemical reactions that generate electricity [8]. Finally, energy generators based on piezoelectricity or triboelectricity are frequently used as energy nanogenerators. Composites made from elastomers, piezoelectric materials, or electrically conductive materials are commonly employed in energy harvesting applications [9].

In addition to energy conversion applications, polymeric materials also play a dominant role in energy storage devices. Frequently used materials include those found in batteries and supercapacitors. In lithium-ion batteries and other types of rechargeable batteries, polymers are used as electrolytes, binders, and separators [10]. For example, solid polymer electrolytes (SPEs) are of great interest because they can improve battery safety by replacing liquid electrolytes, which are often flammable. Moreover, polymer-based electrodes offer flexibility and lightweight properties, making battery systems suitable for portable and wearable electronics [11]. For supercapacitors, energy is stored through the accumulation of electrical charges at the interface between an electrode and an electrolyte. Some conductive polymers, such as polyaniline and polypyrrole, are used in supercapacitor electrodes to increase the device’s charge storage capacity [12]. These materials exhibit high surface area, high conductivity, and robust electrochemical activity. Keeping these aspects in mind, the present editorial summarizes the latest novel articles in the field of polymeric materials for energy conversion and storage. The next section is dedicated to an overview of the latest articles, summarizing the prospects of different polymeric materials for energy applications.

## 2. Overview of Published Articles

Alam et al. [13] presented an energy harvesting system using rubber composites based on silicone rubber, nano-carbon black (NCB), and molybdenum disulfide (MoS_2_). The results reported that at a 17:3 NCB to MoS_2_ ratio, the fracture toughness improved by 184% and elongation at break increased by 93% compared to a sample with 20 phr of NCB as the only filler. The results further indicated that the hybrid filler (17:3 NCB to MoS_2_ ratio) exhibited an over 100% higher output voltage, reaching up to 3.2 mV compared to NCB as the only filler at 20 phr. Jeżowski et al. [14] developed a robust biopolymer membrane for green electrochemical devices. The results showed that a capacitance of 30 F/g, a resistance of 3 Ω, and a voltage of approximately 1.6 V were achieved. These results support the use of this biopolymer membrane as a sustainable alternative for high-performance storage devices. In another study, Guo et al. [15] reported an interesting concept involving redox-active polymers for capacitors as energy storage devices. Automatic sequential polymerization equipment was used to synthesize redox-active polymers on gold electrodes. The total charge generated varied from 2.17 to 7.14 × 10^−4^ C. Alam et al. [16] fabricated high-performance composites based on natural rubber, diatomaceous earth, and carbon nanotubes. The results demonstrated robust performance after adding these reinforcing fillers. For example, the fracture toughness was 9.74 MJ/m^3^ for the unfilled sample and increased by 484% to 56.86 MJ/m^3^ at the 40 phr hybrid sample. The electrical conductivity was around 1.75 × 10^−6^ S/m, and the output voltage was approximately 25 mV for the composite containing 3 phr CNT.

Zappia et al. [17] fabricated composites based on silver nanoparticles for possible water-processable anodes. The results showed that the viscosity of the composites changed from 14.1 to 0.2 Pa·s. The composites also exhibited remarkable photovoltaic properties in organic cells. Alshammari et al. [18] presented a study on the dielectric properties of PVA-based polymeric films. The results indicated that the electrical conductivity increased from 0.82 × 10^−9^ S/cm (unfilled) to 6.82 × 10^−9^ S/cm for the composite sample. Moreover, the relaxation time decreased from 14.2 × 10^−5^ s (unfilled) to 6.35 × 10^−5^ s for the composite sample. Another study by Muñoz et al. [19] investigated the electro-mechanical performance of supercapacitors based on PVA and H_3_PO_4_. The results demonstrated that the composites exhibited high electro-mechanical performance, such as 1.4 F/g and 0.961 W/kg. Additionally, robust performance was reported, with the fabricated device able to recover 96.12% of its original capacitance once the external strain was removed. Surisetty et al. [20] demonstrated the aging behavior of high-density polyethylene (HDPE) and polyketone in liquid organic hydrogen carriers. The aging tests showed that the crystallinity of HDPE at 25 °C was 48.6% after 500 h of aging; however, with an increase in temperature to 60 °C for 500 h, the crystallinity decreased to 46.8%. Yuan et al. [21] presented theoretical studies on the electrothermal response of liquid crystal elastomers for high-performance applications, focusing on various aspects of elastomer-based tunable circuits. Another interesting study by Xu et al. [22] investigated flexible actuators fabricated using polymer ionogels, determining their electromechanical aspects theoretically. The results indicated that with changes in modulus, the mechanical displacement decreased while the blocking force increased, both of which are favorable for actuation properties.

Liu et al. [23] fabricated flexible films from electrically conducting PEDOT materials for thermoelectric energy harvesting. The final samples exhibited a high electrical conductivity of 62.91 S/cm, a Seebeck coefficient of 14.43 µV/K, and a power factor of 1.32 µW/m·K^2^. Tameev et al. [24] reported the charge carrier mobility of poly-TPD-based composites with electron acceptor molecules. The electron and hole mobilities were lower for poly-TPD composites and higher for poly-TPD:PCBM. For example, the electronic mobility was approximately 4.2 × 10^−6^ cm^2^/V·s for poly-TPD and as high as 8.3 × 10^−6^ cm^2^/V·s for poly-TPD:PCBM. In another work, Cho et al. [25] prepared hydrogel electrolytes in rechargeable batteries based on Zn-MnO_2_. They reported very interesting results, such as the battery’s high durability, with negligible capacity loss from approximately 0.4 Ah/g at the 100th cycle to about 0.38 Ah/g at the 800th cycle. Similarly, Hrostea et al. [26] studied the influence of phase change materials as a third component on the optoelectric properties of organic blends. The best sample exhibited superior absorption of 1.07 × 10^5^ cm^−1^ at a wavelength of 628 nm. Moreover, a carrier mobility of 1.41 × 10^−4^ cm^2^/V·s was reported. Lastly, Wei et al. [27] reported a theoretical study of light actuation facilitated by a crystal elastomer fiber. The results were promising, with the elastic coefficient improving by 0.25% and light intensity showing a 20.88% increase. This study is useful for non-circular curved tracks, offering improved adaptability and versatility.

## 3. Summary and Future Outlook

Energy devices based on polymeric materials hold tremendous potential for the future of energy conversion and storage technologies. Continuous innovations in polymer chemistry and materials science are driving the development of new polymer-based systems with enhanced performance [28]. Moreover, the future of flexible and stretchable energy devices, sustainable polymers, and multifunctional materials is bright. For example, stretchable devices made from polymeric materials enable the development of energy systems that can be integrated into clothing, medical devices, and flexible electronics. Additionally, sustainable polymers are being prioritized for the development of biodegradable or recyclable polymeric materials for energy applications [29]. Finally, next-generation materials with diverse functionalities hold promise. For instance, these multifunctional materials can combine energy storage and structural properties within a single material, making them ideal for applications like electric vehicles and smart grids [30]. Overall, the versatility of polymeric materials can be enhanced by ongoing advancements in materials science. These novel materials position themselves as key enablers for next-generation energy technologies, leading to the evolution of new devices that offer more efficient, flexible, and sustainable energy solutions.
